# Estimated Time for Occurrence of Smoking-Related Consequences among Pregnant and Non-Pregnant Women

**DOI:** 10.3390/ijerph6051665

**Published:** 2009-05-15

**Authors:** Monica Ortendahl, Alf Uttermalm, Bo Simonsson, Per Näsman, Tuula Wallsten

**Affiliations:** 1 Center for Safety Research, Royal Institute of Technology, Stockholm, Sweden, Teknikringen 78B, 10044 Stockholm, Sweden; 2 Swedish Social Insurance Agency, Box 802, 721 22 Västerås, Sweden; E-Mail: alf.uttermalm@telia.com; 3 Center for Clinical Research, University of Uppsala, Central Hospital, SE-721 89 Västerås, Sweden, and Karolinska Institute, Department of International Health, SE-171 77 Sweden; E-Mails: bo.simonsson@ltv.se (S.B.); tuula.wallsten@ltv.se (W.T.); 4 Center for Safety Research, Royal Institute of Technology, Stockholm, Sweden; E-Mail: per.nasman@nasman.com

**Keywords:** smoking, pregnancy, quitting, consequences

## Abstract

**Objectives:**

To study time estimates by women smokers for when smoking-related consequences will occur given continuing or quitting smoking. The relationship of these estimates to pregnancy and intent to quit smoking was also investigated.

**Methods:**

Over a two-week period, eighty women, selected to constitute four subgroups formed by pregnant vs. non-pregnant and trying vs. not trying to quit smoking, rated times at which they would expect smoking-related consequences to occur given continuing or quitting smoking.

**Results:**

Somatic health consequences were estimated to occur later than consequences related to mood and social relations. All consequences were estimated to occur later given quitting smoking. Pregnancy had an effect on the estimated time that consequences would occur, with pregnant women estimating earlier occurrence of consequences related to mood and social relations than non-pregnant women did.

**Conclusion:**

Health messages should stress consequences for somatic health in quitting smoking, since outcomes later in time might have too low a value to exert a positive effect on decisions to quit smoking.

## 1. Introduction

Research over the years has clearly established both short- and long-term benefits for women who do not smoke during pregnancy. Smoking in early gestation and through pregnancy is associated with adverse pregnancy outcomes like preterm delivery, low birth weight and stillbirth [1,2].

In recent research time-related aspects of smoking have attracted increased interest, and the possibility of counseling especially tailored to the motivational stage of pregnant women has been explored [3]. Stages of readiness for behavior change (i.e., quitting smoking) are identified in the form of five groups: relapsers, persons in the precontemplation stage, persons in the contemplation stage, actors and maintainers. Readiness has been found to vary with stages of change [4], and persons with a positive attitude towards smoking cessation more often intend to quit smoking [5].

In some respects, addictive behavior could be construed as a temporal preference in which the immediate pleasure of the addictive behavior is preferred to the cessation of the addictive behavior [6]. There is a time-discounting effect in addictive behavior; that is, the value of future consequences diminishes as a function of temporal distance, an effect that represents the weight an individual places on the future relative to the present when she makes a decision that has future consequences [7]. The situation could be described as a choice between the immediate pleasure of having a cigarette, and having good health or avoiding illnesses related to smoking in the remote future.

Impulsivity has been related to perceptions of positive and negative reinforcement from smoking [8], and it is supposed that those who value remote values less compared to those near in time are most impulsive [9]. More impulsiveness has been associated with smoking, and in a study [10] it was found that impulsiveness predicted postpartum relapse to cigarette smoking among pregnant women. Discount rates and impulsiveness have also been found to be higher for current smokers compared to never-before smokers [11].

The purpose of the present study was to examine judgments by pregnant and non-pregnant women who were intending either to quit or to continue smoking, about when they expected smoking-related consequences to occur given continuing or quitting smoking. Because failures in quitting have been attributed to a shortened time perspective, changes in perceived time of outcome for different consequences related to smoking were of interest. In studying time discounting and patterns of cigarette smoking, discount rates have generally been obtained by the discounting of monetary rewards [12].

In the present study pregnant and non-pregnant women’s judgments of times at which smoking-related consequences would occur were studied over a span of fourteen days. Women who expressed an intention to quit smoking and women who did not intend to quit smoking were studied, thus constituting four subgroups defined by pregnancy/non-pregnancy and intent to quit/no intent to quit.

## 2. Materials and Methods

### 2.1. Participants

Forty pregnant women were contacted in family practices in Bulgaria when they were under ordinary medical observation during pregnancy. Their pregnancies were normal and uncomplicated. Forty non-pregnant women were recruited at the same clinics when they came for regular examinations. The women were asked whether they smoked. If they smoked they were asked about their intent to quit or not quit smoking. The women were given the question “Do you intend to quit smoking?” “Yes” was interpreted as an intention to quit. Of course, it could be argued that time does matter with regard to intent to quit. For example, it would be a difference between someone who was intending to quit in ′the near future′ versus ′someday′.

Depending on whether or not they intended to quit smoking, they were assigned to one of four groups. Each group consisted of twenty women: pregnant smokers who did not intend to quit smoking, pregnant smokers who intended to quit smoking, non-pregnant women who did not intend to quit smoking, and non-pregnant women who intended to quit smoking. Approval for the study was obtained from the institutional review board. No intervention was used to try to convince participating women not to smoke. All women agreed to participate in the study and gave informed consent in writing. The women received no compensation for participating.

### 2.2. Procedure

A questionnaire was given individually to the women for each consecutive day of the first week of the study, with a final questionnaire two weeks after the start of the study. All the questions were personally administered to the eighty women over a fourteen-day period. The distribution of survey time points was chosen to provide close follow-up during the first week, as many changes in attitudes and intent to quit have been found to occur during this period [13]. The final survey point after two weeks of participation was chosen in order to note changes over a somewhat longer period.

The smoking-related health consequences presented in the questionnaire were selected to cover positive and negative aspects involved in continuing to smoke and in quitting smoking. The time each consequence was judged likely to occur was rated for both conditions of continuing and quitting smoking. The consequences, presented in Table 1, were related to somatic health and to mood and social relations; pregnant women rated an additional nine consequences related to smoking and pregnancy.

A seven-point Likert scale was used, with 1 = this week, 2 = in 1–2 weeks, 3 = in 3–4 weeks, 4 = in 1–6 months, 5 = in 7–12 months, 6 = in 13 months or later, and 7 = never.

The women were instructed to respond to each questionnaire in the early evening (about 6 p.m.), and not to think back to questionnaires they had already filled in, but to answer based on their present state of mind. The short distance between the questionnaires might seem inconsequential. However, as earlier mentioned, distribution of survey time points was chosen to provide close follow-up during the first week, as many changes in attitudes and intent to quit have been found to occur during this period [13]. On day 1 the women were asked about background information including demographics. Then, over the course of the fourteen-day period (i.e., days 1–7 and day 14), all women made ratings of the time at which they estimated the various consequences would occur.

### 2.3. Statistical Methods and Data Management

Repeated measurements analysis was used to analyze time-dependent data, and multiple comparisons of continuous data were performed by analysis of variance. The procedure proposed by Fisher was used to control for multiplicity [14]. In order to evaluate hypotheses of variables in contingency tables, the chi-square test was used or, in the case of small expected frequencies, Fisher’s Exact Test. The Kruskal-Wallis non-parametric one-way analysis of variance test was used in simultaneously comparing results from all four groups.

In addition, descriptive statistics and graphical methods were used to characterize the data [14]. The study employs multiple hypotheses testing, where each hypothesis was analyzed separately, and the existence of patterns in and the consistency of the results were considered in the analysis. All analyses were carried out using the SAS system 8.02 for Windows, and the 5, 1 and 0.1% levels of statistical significance were considered. In the case of a statistically significant result the probability value (p-value) is given.

## 3. Results

### 3.1. Specification of the Sample

Table 2 presents background data regarding age, number of years of smoking, number of earlier attempts to quit smoking and number of cigarettes smoked per day.

There was a statistically significant difference (p = 0.003) between the four groups for number of earlier attempts to quit smoking. Women intending to quit smoking had tried to quit more often in the past, with means 2.4 and 4.0 for pregnant and non-pregnant women, respectively, compared with women not intending to quit smoking (means 1.8 and 1.7). No other statistically significant differences were obtained. The mean age of the women was 25.1 years. They had been smokers, on average, for 6.5 years, and mean number of earlier attempts to quit smoking was 2.5. They smoked an average of 10.5 cigarettes per day. All women had a high school education.

The mean number of pregnancy months was 4.8 (range, 2–8 months) for women intending to quit smoking and 4.9 (range, 2–9 months) for women not intending to quit smoking. No statistically significant difference was obtained (Mann-Whitney test p = 0.93) in number of pregnancy months between women intending to quit and not intending to quit smoking.

### 3.2. Estimated Time for Consequences to Occur Given Continuing or Quitting Smoking

Repeated analyses of variance (ANOVAs), with quitting smoking and pregnancy as the between-subjects variables and days as the within-subjects variable, are presented in Table 3. The ANOVAs were performed separately for somatic health consequences, consequences related to mood and social relations, and consequences related to pregnancy.

**Figure 1.**
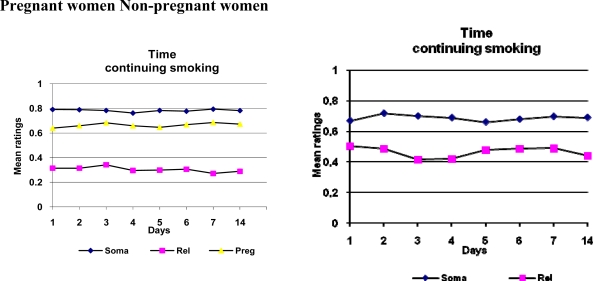
Ratings over the two-week period for smoking-related consequences to occur for pregnant and non-pregnant women given the condition of continuing smoking.

ANOVAs were performed for variables of pregnant, quitting, days, pregnant*quitting, pregnant*days, quitting*days and pregnant*quitting*days for ratings of time for the consequences to occur given continuing and quitting smoking. Significant differences were revealed by the ANOVAs for being pregnant or not, for consequences related to mood and social relations; this applied both to the condition of continuing and of quitting smoking. The intention to quit or not had an effect on consequences related to mood and social relations and on somatic health consequences. There was also an effect on judgments over days for consequences related to pregnancy. Mean ratings over time for pregnant and non-pregnant women of estimated occurrence of consequences given continuing or quitting smoking are displayed in Figure 1.

The estimated time for the consequences to occur showed a little variation over time for both conditions of continuing and of quitting smoking. For both conditions, of continuing and of quitting, mood and social relations were judged to be affected first, after which consequences related to pregnancy and somatic health consequences were expected to appear. This applied to all women. For both conditions, of continuing and of quitting, pregnant women estimated consequences related to mood and social relations to occur earlier than non-pregnant women did.

All consequences of smoking, for somatic health, mood and social relations, and pregnancy, were estimated to occur later given the condition of quitting smoking. Pregnant women estimated that consequences related to mood and social relations would occur in approximately 3–4 weeks given the condition of continuing to smoke, and in 1–6 months for the condition of quitting. Non-pregnant women estimated the time for these consequences to occur as 1–6 months and 7–12 months, respectively.

Pregnant women estimated that the somatic health consequences would occur in somewhat less than 13 months or later given the condition of continuing to smoke, and in less than 13 months or later given the condition of quitting. Non-pregnant women estimated the times at 7–12 months and in 13 months or later, respectively. Pregnant women estimated that the consequences related to pregnancy would occur in 3–4 weeks given the condition of continuing to smoke, and in 1–6 months given the condition of quitting.

## 4. Discussion

In general, smoking-related consequences related to mood and social relations were estimated to occur sooner than pregnancy-related and somatic health consequences. The difference between estimated occurrence of somatic health consequences and consequences related to pregnancy was very small. All consequences were estimated to occur later given the condition of quitting smoking.

Pregnancy had an effect on the time at which the consequences were estimated to occur. Pregnant women estimated that consequences related to mood and social relations would occur earlier than nonpregnant women did; this was so both for the conditions of continuing to smoke and of quitting. The intent to quit showed an effect in women’s estimates of when the somatic consequences of smoking would appear given the condition of continuing to smoke; it also showed in expected consequences for mood and social relations given the condition of quitting.

Intent to quit smoking was related to the number of earlier attempts to quit. This finding suggests that the women who are trying to quit smoking are generally more motivated to stop smoking than those who are not trying to stop. It indicates that success is preceded by a certain number of attempts to quit, and that the chance is improved by every attempt. The result is in accord with earlier results obtained by Pickett, Wakschlag, Dai and Leventhal [15], where a substantial proportion of the women studied exhibited a pattern of repeated cessation and relapse. However, in our study the number of participants was quite small with a small number of successful quitters. Many attempts to quit are unprepared [16]; this suggests that models of smoking cessation should place greater emphasis on the dynamic nature of motivation to quit.

Of course, there is an increased statistical probability of success, but there is also an increased psychological probability. In addition, non-pregnant women who were trying to quit and who had smoked for the longest period (8.7 years) also exhibited the highest number of previous attempts to quit (4.0). It is expected that the number of attempts is related to length of time smoking.

A new feature of the present design was that the women performed ratings at the start of the study and throughout the period of the study. The current design made it possible to obtain information about changes over time when processes that lead to success in quitting smoking or to relapse are being studied. In fact, a distinction must be made between anticipated waiting time and actual waiting time: the actual time that elapses between the present and a future event, positive or negative, is evidently more aversive.

Our data support the potentials of the new framework introduced in the present study in analyzing addictive behavior and in smoking cessation programs. The present results indicate that discounting could be expected to have the largest impact on somatic health consequences in that they were estimated to occur later [17]. Smokers generally exhibit rapid loss of value for delayed health outcomes; this underscores the need for smoking-cessation treatments that provide relatively immediate consequences for abstinence.

Therefore, health messages should stress the outcome in somatic health consequences for quitting smoking, as effects later in time could have a low value and thereby a negative affect on decisions to quit smoking. Another factor that health messages should emphasize is that consequences on somatic health are more likely to be irreversible than effects on relationships.

For the two conditions of continuing to smoke and quitting smoking, there were only small changes over time, suggesting that this variable is independent of the passage of time. One possible explanation is that the judgments encompass a time span from this week to 13 months or later and in its extreme, never. The duration of the study, however, was only fourteen days.

Smoking is probably most salient in pregnant women because women who smoke during pregnancy subject themselves and their developing fetus and newborn to special risks. Although evidence indicates that individual smoking cessation counseling does not increase quitting rates during pregnancy, the method may have long-term effects [18]. It is therefore important to identify variables which ensure that the initial attempts to quit are translated into long-term abstinence [19,20].

Smoking during pregnancy is a complex and variable behavior for many women, and brief smoking cessation interventions in early pregnancy are likely to be inadequate for many smokers during pregnancy. It has been suggested [21] that differing predictors may contribute to understanding the different transitional stages of smoking cessation. Of course, the etiology for smoking like e.g., experiences of violence and stress may impact the findings.

Problems related to behavior change and its maintenance are a significant challenge to domains that deal with health-related behaviors, as professional health care services do. This applies to addictive habits like smoking; most cessation programs are generally effective only temporarily in helping people with their struggle against smoking [22].

The prevalence of smoking is high in Bulgaria [23], and results of the present study may be limited to countries with similar smoking habits. High prevalence of smoking has been found to be associated with a lower motivation to quit smoking, fewer attempts to quit and higher cigarette consumption among smokers [24]. A recent study [25] found that only 68% of smokers were asked about their smoking habits during pregnancy by their gynecologist, and that both smokers and non-smokers had insufficient information about the impact of smoking on their baby.

## 5. Conclusions

All women estimated that the condition of quitting smoking would make consequences occur later. Pregnant women estimated that consequences related to mood and social relations would occur earlier than non-pregnant women did. The period of pregnancy might provide a window of opportunity to promote smoking cessation and smoke-free families.

Further, the significance of the value of remote outcomes in explaining addictive behavior should be considered to a greater extent than it has been in the past, as it is evident that such behavior is not the result of one single action but a long series of actions that have been taken [26,27]. The study illustrates the necessity of considering changes that occur over time when we are explaining health behavior. The present study encompassed two weeks, and future studies may benefit from studying a longer period of time.

## Figures and Tables

**Figure 2.**
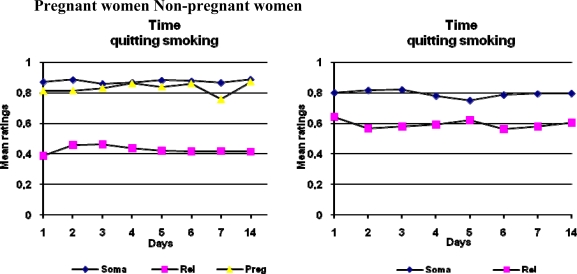
Ratings over the two-week period for smoking-related consequences to occur for pregnant and non-pregnant women given the condition of quitting smoking.

**Table 1.** Health consequences rated with regard to value and probability.

***Somatic health consequences***
Your physical condition becomes worse
You are afflicted with heart disease
You feel pain in your chest
You get lung cancer
Your life expectancy becomes shorter
You lose some of your physical strength
***Consequences related to mood and social relations***
Others like you
You have good relations with others
You feel free to do whatever you wish
Your self-confidence improves
You satisfy others who worry about your health
You feel pleasure and satisfaction
***Consequences related to pregnancy***
You worry about the future health of your unborn child
You influence the condition of your fetus in a negative manner
Your home environment is unhealthy for children
Your own health during pregnancy is at risk
Your home environment can cause allergy in children
Your child will become a smoker
You have a miscarriage
You have a child with a low birth weight
You have a difficult child delivery

**Table 2.** Demographics based on age, number of years of smoking, number of earlier attempts to quit smoking and number of cigarettes per day for the four groups (n=80).

	**Mean**	**SD**	**Median**	**Range**
**Pregnant women not intending to quit (n=20)**				
Age	24.1	3.28	24.0	20–30
Number of years of smoking	5.1	3.15	4.5	1–11
Number of earlier attempts	1.8	2.63	1.0	0–10
**Pregnant women intending to quit *(n=20)***				
Age	25.2	3.96	24.5	20–31

**Number of years of smoking**	5.8	2.51	5.0	3–13

Number of earlier attempts	2.4	2.22	2.0	0–10
Number of cigarettes per day	11.2	4.66	10.0	4–20
**Non-pregnant women not intending to quit *(n=20)***				
Age	26.7	3.86	27.5	20–31
Number of years of smoking	8.7	4.22	9.5	2–16
Number of earlier attempts	4.0	2.96	3.5	0–12
Number of cigarettes per day	10.2	3.86	10.0	4–20
***Total* (n=80)**				
Age	25.1	3.79	25.0	20–31
Number of years of smoking	6.5	3.51	6.0	1–16
Number of earlier attempts	2.5	2.52	2.0	0–12
Number of cigarettes per day	10.5	4.58	10.0	4–20

**Kruskal-Wallis test for group differences**	**Chi-Square**		**df**	**p-value**

Age	4.805		3	0.187

Number of years of smoking	7.223		3	0.065
Number of earlier attempts	13.797		3	0.003**
Number of cigarettes per day	2.429		3	0.488

* p < 0.05

** p < 0.01

*** p < 0.001

**Table 3.** ANOVA with repeated measures for variables of pregnancy/non-pregnancy, quitting/non-quitting and days. Ratings of time for the consequences to occur given continuing and quitting smoking.

	*Somatic health*		*Mood and social relations*		*Pregnancy*	

	**F-value**	**Probability**	**F-value**	**Probability**	**F-value**	**Probability**
**TIME**						
**continuing smoking**						
PREGNANT	2.19	0.14	8.16	0.0055*	–	–
QUITTING	4.41	0.0391*	0.07	0.79	0.77	0.38
PREGNANT*QUITTING	0.51	0.83	0.14	0.99	12.45	0.0001*
DAYS	0.51	0.83	0.14	0.99	12.45	0.0001*
PREGNANT*QUITTING	0.12	0.74	0.17	0.68	–	–
PREGNANT*DAYS	0.71	0.67	0.63	0.73	–	–
QUITTING*DAYS	0.75	0.63	1.20	0.30	1.94	0.06
**TIME**						
**quitting smoking**						
PREGNANT	0.50	0.48	9.03	0.0036*	–	–
QUITTING	0.03	0.86	5.21	0.0252*	0.08	0.78
DAYS	0.47	0.86	0.73	0.65	2.35	0.0240
PREGNANT*QUITTING	0.45	0.50	0.15	0.70	–	–
PREGNANT*DAYS	1.01	0.42	1.20	0.30	–	–
QUITTING*DAYS	0.92	0.49	1.43	0.19	2.18	0.359*
PREGNANT*QUITTING*DAYS	1.19	0.30	0.46	0.86	–	–

PREGNANT, QUITTING, and PREGNANT*QUITTING based on df=1

DAYS, PREGNANT*DAYS, QUITTING*DAYS, and PREGNANT*QUITTING*DAYS based on df=7;

* p=0.05; Data in boldface are significant at p < 0.05
